# Identification of Genes Involved in Indole-3-Acetic Acid Biosynthesis by *Gluconacetobacter diazotrophicus* PAL5 Strain Using Transposon Mutagenesis

**DOI:** 10.3389/fmicb.2016.01572

**Published:** 2016-10-07

**Authors:** Elisete P. Rodrigues, Cleiton de Paula Soares, Patrícia G. Galvão, Eddie L. Imada, Jean L. Simões-Araújo, Luc F. M. Rouws, André L. M. de Oliveira, Márcia S. Vidal, José I. Baldani

**Affiliations:** ^1^Laboratório de Genética de Microrganismos, Departamento de Biologia, Universidade Estadual de LondrinaLondrina, Brazil; ^2^Embrapa AgrobiologiaSeropédica, Brazil; ^3^Laboratório de Bioquímica Molecular, Departamento de Bioquímica e Biotecnologia, Universidade Estadual de LondrinaLondrina, Brazil

**Keywords:** diazotrophic rhizobacteria, endophyte, phytohormone, plant-microbe interaction, auxin, L-amino acid oxidase

## Abstract

*Gluconacetobacter diazotrophicus* is a beneficial nitrogen-fixing endophyte found in association with sugarcane plants and other important crops. Beneficial effects of *G. diazotrophicus* on sugarcane growth and productivity have been attributed to biological nitrogen fixation process and production of phytohormones especially indole-3-acetic acid (IAA); however, information about the biosynthesis and function of IAA in *G. diazotrophicus* is still scarce. Therefore, the aim of this work was to identify genes and pathways involved in IAA biosynthesis in this bacterium. In our study, the screening of two independent Tn5 mutant libraries of PAL5^T^ strain using the Salkowski colorimetric assay revealed two mutants (Gdiaa34 and Gdiaa01), which exhibited 95% less indolic compounds than the parental strain when grown in LGIP medium supplemented with L-tryptophan. HPLC chromatograms of the wild-type strain revealed the presence of IAA and of the biosynthetic intermediates indole-3-pyruvic acid (IPyA) and indole-3-lactate (ILA). In contrast, the HPLC profiles of both mutants showed no IAA but only a large peak of non-metabolized tryptophan and low levels of IPyA and ILA were detected. Molecular characterization revealed that Gdiaa01 and Gdiaa34 mutants had unique Tn5 insertions at different sites within the GDI2456 open read frame, which is predicted to encode a L-amino acid oxidase (LAAO). GDI2456 (*lao* gene) forms a cluster with GDI2455 and GDI2454 ORFs, which are predicted to encode a cytochrome C and an RidA protein, respectively. RT-qPCR showed that transcript levels of *lao. ccc*A, and *rid*A genes were reduced in the Gdiaa01 as compared to PAL5^T^. In addition, rice plants inoculated with Gdiaa01 showed significantly smaller root development (length, surface area, number of forks and tips) than those plants inoculated with PAL5^T^. In conclusion, our study demonstrated that *G. diazotrophicus* PAL5^T^ produces IAA via the IPyA pathway in cultures supplemented with tryptophan and provides evidence for the involvement of an L-amino acid oxidase gene cluster in the biosynthesis of IAA. Furthermore, we showed that the mutant strains with reduction in IAA biosynthesis ability, in consequence of the lower transcription levels of genes of the *lao* cluster, had remarkable effects on development of rice roots.

## Introduction

*Gluconacetobacter diazotrophicus* is a nitrogen-fixing endophytic bacterium found colonizing the interior of roots and stems of sugarcane plants (*Saccharum officinarum* L; Cavalcante and Döbereiner, 1988; Gillis et al., 1989; James et al., 1994) and also other crops such as sweet potato, pineapple, coffee, elephant grass, and rice (Jimenez-Salgado et al., 1997; Tapia-Hernández et al., 2000; Muthukumarasamy et al., 2005; Saravanan et al., 2008; Rouws et al., 2010). Recently, internal tissue colonization of *Arabidopsis thaliana* and sorghum genotypes by *G. diazotrophicus* was also reported (Rangel de Souza et al., 2016; Yoon et al., 2016). *G. diazotrophicus* has been used as an endophytic model organism to evaluate plant-bacterial interactions with non-legume host (Saravanan et al., 2008). The genome sequence of *G. diazotrophicus* PAL5^T^ strain was earlier determined providing important insights into metabolism, nitrogen fixation regulation, endophytic relationship, and other processes, including phytohormone production (Bertalan et al., 2009). *G. diazotrophicus* strains produce plant hormones including gibberellins, cytokinins, and auxins (Fuentes-Ramirez et al., 1993; Bastián et al., 1998). Although the function of auxins in the *G. diazotrophicus*–sugarcane interaction is not well understood, inoculation studies have suggested that bacterial auxin promotes growth of sugarcane in conjunction with biological nitrogen fixation (BNF) (Sevilla et al., 2001; Oliveira et al., 2002; Muñoz-Rojas and Caballero-Mellado, 2003; Lee et al., 2004). In many plant growth-promoting rhizobacteria, auxin has been reported to stimulate the formation of lateral roots and root hairs, thus increasing total root surface and leading to an enhanced mineral uptake (Sukumar et al., 2013; Verbon and Liberman, 2016). Additionally, auxin functions as a signaling molecule that participates in gene regulation in some bacteria and as an effector molecule in plant–microbe interactions (Spaepen et al., 2007; Spaepen and Vanderleyden, 2011; Patten et al., 2013).

Five tryptophan-dependent pathways for indole-3-acetic acid (IAA) biosynthesis have been described in bacteria, which are named according to their intermediates: indole-3-pyruvate (IPyA), indole-3-acetamide (IAM), indole-3-acetonitrile (IAN), tryptamine (TAM), and tryptophan side chain oxidase (TSO; Spaepen and Vanderleyden, 2011; Patten et al., 2013). Earlier studies have suggested that *G. diazotrophicus* produces IAA via the IPyA pathway (Fuentes-Ramirez et al., 1993; Lee et al., 2004), a common pathway found in various plant growth-promoting rhizobacteria; however, the genes or enzymes involved have not yet been identified. Interestingly, Lee et al. (2004) reported that mutations in genes of cytochrome C maturation resulted in strains capable of producing only 5% of the auxin produced by the wild-type strain revealing that a cytochrome C protein may be involved in the production of IAA by *G. diazotrophicus*.

The use of Tn5-mediated mutagenesis and the consecutive screening of the resulting mutant libraries has been broadly used to identify key genes involved with complex bacterial functions, as those needed for a compatible plant-endophyte interaction. Here, we report the screening of 5,700 mutants from two random and independent Tn5 mutant libraries from *G. diazotrophicus* PAL5^T^strain, aiming to identify genes and pathways involved in the IAA biosynthesis. When bacteria were grown in culture media under tryptophan supplementation and further analyzed using the Salkowski colorimetric assay for indolic compounds, two mutants were identified as largely impaired in the ability to convert tryptophan to indolic compounds. Further characterization of both mutants revealed a single insertion of the Tn5 transposon in each mutant, although the insertions occurred at different positions within the same gene, which is predicted to encode an L-amino acid oxidase protein. The functional characterization of these mutants enabled the identification of an L-amino acid oxidase gene cluster involved in the biosynthesis of IAA by this bacterium and showed that the integrity of this cluster is important to promote growth of rice roots.

## Material and Methods

### Bacteria Strain and Culture Media

*Gluconacetobacter diazotrophicus* strain PAL5^T^ (BR11281^T^) was obtained from the Diazotrophic Bacteria Culture Collection of Embrapa Agrobiology (CNPAB, Rio de Janeiro, Brazil) and grown in LGIP (Cavalcante and Döbereiner, 1988) or DYGS (Rodrigues Neto et al., 1986) culture media. For IAA quantification tests and expression analyses, was used LGIP broth without bromothymol blue and supplemented with 10 mM (NH_4_)_2_SO_4_. Antibiotic kanamycin (200 μg.mL^-1^) was used when required. Amino acid tryptophan (100 μg.mL^-1^) was used as IAA precursor, except in tryptophan free treatments.

### Transposon Mutagenesis and Library Screening for IAA-Deficient Mutants

Two independent transposon libraries of *G. diazotrophicus* PAL5^T^ were generated using the EZ-Tn5^TM^ < KAN-2 > Tnp Transposome^TM^ system as described previously (Rouws et al., 2008). The transposon mutant libraries, which consisted of 5,700 mutants (3,000 of the first mutagenesis experiment and 2,700 of the second), were grown in 1 mL of LGIP medium with tryptophan in 96-well microplates for 48 h at 30°C and 200 rpm for screening of mutant strains altered in production of indolic compounds. LGIP medium non-inoculated or inoculated with PAL5^T^ strain were used as blank and positive control, respectively. Optical density at 620 nm was determined on spectrophotometer Labsystems iEMS Reader MF. The remaining culture was centrifuged (3,220 × *g*, 15 min) and then 150 μL of the cell-free supernatants were mixed with 100 μL of Salkowski reagent (1 mL of 0,5 M FeCl_3_ + 50 mL 35% HClO_4_), according a previously described method (Sarwar and Kremer, 1995). After 30 min of incubation in the dark, the absorbance was measured at 540 nm and the concentration of indolic compounds was determined using a calibration curve of 0 a 80 μg.mL^-1^ of IAA (Sigma-Aldrich) as standard. Mutants that displayed lower production (≤50%) of indolic compounds relative to PAL5^T^ strain were selected for confirmation assay, in which the mutants were grown in 50 mL of LGIP medium in Erlenmeyer flasks at 30°C and 150 rpm and after 48, 72, and 96 h of incubation, the Salkowski assay was performed for indolic compounds quantification. After the confirmation assay, mutants which showed ≤95% of indolic compounds production relative to the type strain were selected and further analyzed.

### Quantification of Growth and Indolic Compounds Production

Bacteria were grown as pre-inoculum in 10 mL of LGIP medium for 48 h, and 1 mL of these pre-cultures (optical density at 600 nm equal 1.0) were inoculated in 1 L Erlenmeyer flasks containing 300 mL of LGIP medium with or without 100 μg.mL^-1^ of tryptophan; three biological replicates were realized. Non-inoculated or PAL5^T^ inoculated LGIP medium were used as blank and positive control, respectively. Cultures were incubated at 30°C and 200 rpm and growth was monitored by measuring optical density at 600 nm at time intervals of 12, 24, 38, 48, 60, and 84 h of growth. Cells were harvested by centrifugation and supernatants were used to determine the presence of indolic compounds by the Salkowski assay. Pellet cells were lysed (NaOH 0,1 M, 15 min, 90°C) and total protein content was determined by Bradford reaction at 595 nm, using a bovine serum albumin calibration curve of 0 a 40 μg.mL^-1^ as standard (Bradford, 1976).

### High Performance Liquid Chromatography (HPLC) Analysis

Cultures grown in LGIP medium with or without L-tryptophan were analyzed by HPLC to identify and quantify the indolic compounds produced. Samples of 20 mL were collected at the exponential (16 h) and stationary (60 h) phases and centrifuged (8,228 × *g*; 4°C; 10 min). Cell-free supernatants were loaded in a solid phase cartridge (Strata-X), which was previously activated with methanol and equilibrated with 0.1 M phosphate buffer pH 7.0. Indolic compounds were eluted from cartridge with 10 mL of methanol, which was removed by evaporation *in vacuo* (Centrivap concentrator, Labconco) at 37°C. The compounds were dissolved in 1 mL of methanol and 50 μL was analyzed using a LC10-A manifold (Shimadzu) and a Luna C18 column (30 cm × 3.9 mm, 5 μm, 100 Å, Phenomenex). The mobile phase was a gradient of phosphate buffer (pH 7.0) and methanol (20–60%) for 40 min pumped at flow rate of 0.5 mL/min. Compounds were detected by ultraviolet absorbance at 254 nm. Identification and quantification was based, respectively, on retention time and the calibration curve of the following compounds: tryptophan, indole-3-acetate, indole-3-lactate (ILA), IAM, IPyA, IAN, tryptamine, anthranilate, indole-3-propionate, indole-3-ethanol, and indole (MP Biomedicals and Sigma Aldrich).

### Identification of Transposon Insertion Site

Total DNA isolation and gel electrophoresis were performed as previously described (Sambrook and Russell, 2001). Southern blot and inverse PCR (iPCR) were performed as previously reported (Goryshin et al., 2000; Rouws et al., 2008). Briefly, DNA (1 μg) was digested with *Pst*I, *Eco*RI, or *Eco*RV enzymes and hybridized against a *Bam*HI-*Xho*I fragment of EZ::Tn5 transposon labeled with [α^32^P] dCTP used as probe. For iPCR, DNA digested with *Pst*I or *Pvu*I were treated with T4 DNA ligase to produce self-circularized fragments and then, amplified by PCR (Rouws et al., 2008). iPCR products were electrophoresed, purified, and sequenced on a MegaBace 1000 automated sequencer using a DYEnamic ET terminator sequencing kit, as recommended by the manufacturer (GE Healthcare).

### Sequence Analyses

In order to identify genomic Tn5 insertion sites, the transposon-flanking sequences were locally aligned against the *G. diazotrophicus* PAL5^T^ genome, GenBank accession number NC_010125.1 (Bertalan et al., 2009) using the basic local alignment search tool (BLAST; Altschul et al., 1997). Functional analysis of protein sequences was performed using the InterProScan software v5.17 (Zdobnov and Apweiler, 2001; Mitchell et al., 2015) to predict the presence of conserved domains and other functional domains such as signal peptides. Transmembrane motifs were predicting with the TopPred tool (von Heijne, 1992). Genomic context analysis and operon prediction were performed with the Artemis genome browser (Rutherford et al., 2000), Rhizobase (Fujisawa et al., 2014), and OperonDB database (Pertea et al., 2009). Comparative analyses using ACT (Carver et al., 2005) were used to assess the conservation of the gene region in other bacterial species. When X-ray crystallography data was available, tertiary structures were predicted and compared with homologous polypeptide sequences with validated protein structure using the I-TASSER server (Yang et al., 2015). Amino acid sequences (**Supplementary Table 1**) were aligned with Clustal Omega (Sievers and Higgins, 2014) and phylogenetic trees were constructed with MEGA 6 (Tamura et al., 2013) using the neighbor joining method (Saitou and Nei, 1987) and the JTT distance matrix (Jones et al., 1992), assuming that rate variation among sites followed a gamma distribution. The shape parameter (a) of the gamma distribution was estimated by the TREE-PUZZLE program (Schmidt et al., 2002). The validity of branching patterns was assessed by bootstrapping (Felsenstein, 1985) using 1,000 iterations.

**Table 1:** Sequences of primers used in RT-qPCR analyses of *Gluconacetobacter diazotrophicus* strains.

Gene (ORF ID)	Primer sequences (5′ – 3′)	Positions^a^	Length (bp^b^)
*lao* (GDI2456)	F – GTATCCCAGCCACGGCTATTTC	1209–1360	152
	R – GATAGTTCGGATGGATGACCTG		
*ccc*A (GDI2455)	F – GCAATCTACCAGCATGTGTG	121–340	220
	R – AATGGCTGCGGACATAGTTC		
*rid*A (GDI2454)	F – GCCAGCACGATCTATCTGAG	142–355	214
	R – AGTCGATCTTGCCCAGCTTC		
*rpo*D (GDI3335)	F – ACAACGACACCACTCTGCTG	68–198	131
	R – CTCCGACGACATCTGATCCT		


*^a^Nucleotide positions were based on *G. diazotrophicus* genome (GenBank accession number NC_010125.1); ^b^Amplicon sizes.*

### Reverse Transcription Quantitative Real-Time PCR (RT-qPCR)

RT-qPCR analyses were performed using the 7500 Fast Real Timer PCR system and SYBR Green PCR Master Mix (Applied Biosystems), as previously described (Galisa et al., 2012). Primer sets of target genes (*lao. cccA*, and *ridA*) and of the endogenous control gene *rpoD* (**Table 1**) were designed using Primer Express 3.0 (Applied Biosystems, USA). Bacteria were grown for 72 h in LGIP medium with or without L-tryptophan and three biological replicates. Cells (5.5 × 10^7^ cells/mL) were centrifuged (4,000 × *g* for 5 min) and pellet was used for total RNA extraction using the Trizol reagent, as recommended by the manufacturer (Invitrogen). RNA purity and quantity were checked by using a Nanodrop spectrophotometer and by agarose gel electrophoresis. cDNA was synthesized in triplicate using the Superscript^TM^ III Reverse Transcriptase kit, 5 μg of total RNA treated with DNAase I (Invitrogen) and 250 ng of random primers, according to the manufacturer’s instructions (Invitrogen). RT-qPCR was performed in a volume of 15 μL containing 7.5 μL of SYBR Green PCR Master Mix (Applied Biosystems), 10 pmol of each forward and reverse primers (**Table 1**), and 5.0 μL of 1:20-diluted cDNA template. Amplification was performed in a 7500 Fast Real-Time PCR system (Applied Biosystems) using the following thermal cycling conditions: 2 min at 95°C, followed by 40 cycles of 20 s at 95°C and 30 s at 60°C. RT-qPCR assays were carried out with three technical replicates per biological replicate and non-template control was included. Relative expression ratio of target genes in treatment (Gdiaa01) versus control (PAL5^T^) was calculated using REST-MCS^®^ software version 2 (Pfaffl et al., 2002), which applies a mathematic model with primer efficiency correction and 2000 randomizations. Data are expressed as means ± standard errors (SE) from three technical replicates of each biological replicates.

### Plant Growth Promoting Inoculation Effects

The *G. diazotrophicus*–plant interaction experiment was performed with rice seedlings, a recognized host for *G. diazotrophicus* and responsive plant for its inoculation (Muthukumarasamy et al., 2005, 2007; Rouws et al., 2010). Rice seeds of variety IAC4440 were peeled and surface disinfested as described by Hurek et al. (1994). After disinfestation, the seeds were aseptically transferred to plates containing LB medium diluted 10X with 0.5% agar and incubated for 2 days at 37°C in the dark. Germinated seedlings free of microorganisms were immersed for 30 min in saline solution containing (10^5^ CFU.mL^-1^) of *G. diazotrophicus* PAL5^T^ or Gdiaa01 mutant. After this period, plants were transferred to Germitest papers that were disposed in sterile plastic tray with 0.5X Hoagland’s solution (Hoagland and Arnon, 1950). Trays containing the seeds were placed inside plastic bags and incubated at 28°C and photoperiod of 12 h. Rice seedlings were harvested 3 and 7 days after inoculation and the roots were scanned. The images were analyzed using the WinRHIZO software (Regent Instruments Inc), where the length and surface area were measured and the number of forks and root hairs were counted (Arsenault et al., 1995). The experiment was performed with four biological replicates in a completed randomized design. Statistical analyzes were performed using the pairwise *t*-test at *p* < 0.05.

## Results

**FIGURE 1:**
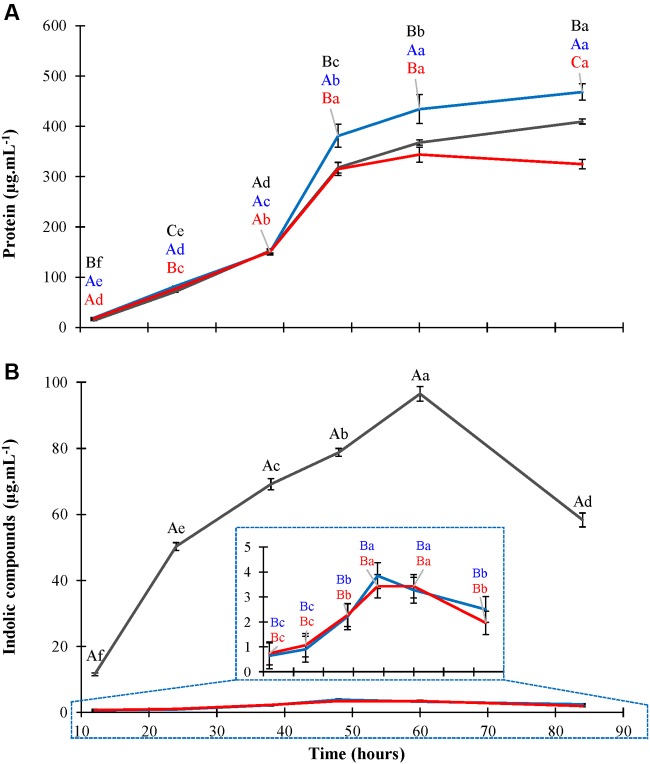
**Growth and production of indolic compounds by *Gluconacetobacter diazotrophicus* PAL5^T^ and mutants.** Bacteria were grown in LGIP medium with 100 μg.mL^-1^ of L-tryptophan and protein **(A)** and indolic compounds production **(B)** were monitored by Bradford and Salkowski assays, respectively. Data represent the mean ± standard error (SE) of three biological replicates. The gray, blue, and red lines represent, respectively, PAL5^T^, Gdiaa01, and Gdiaa34 strains. Indolic compounds production by Gdiaa01 and Gdiaa34 strains is shown in detail inside panel **(B)**. Letters indicate, respectively, statistical significant differences among strains at each harvest point (Capital) and between harvest times for each strain (lower case) according to the Tukey test (*p* ≤ 0.05).

Two libraries containing around 3,000 and 2,700 Tn5 mutants were obtained from independent transformation experiments and screened for low production of indolic compounds during growth in liquid LGIP medium with tryptophan. Among the phenotypes altered in the indolic compound synthesis, two mutants exhibiting strong IAA-deficient phenotypes were isolated: mutant Gdiaa34 obtained from the first round of mutagenesis and Gdiaa01 from the second experiment. These mutants were characterized in more detail. Gdiaa01 and Gdiaa34 mutants were grown in LGIP medium with L-tryptophan and then growth and indolic compounds production was monitored until the stationary phase. Gdiaa01 and Gdiaa34 mutants had growth patterns similar to the wild-type strain and differed only in the late stationary phase, as revealed by protein content in LGIP medium (**Figure 1**). The monitoring of growth over time by optical density at 600 nm also revealed that mutation did not have a remarkable effect on growth of the two mutants (data not shown). In contrast, the production of indolic compounds was significantly reduced in both mutants and statistically differed from the wild-type within 12 h after inoculation (**Figure 1**). PAL5^T^ strain produced 11.5 μg.mL^-1^ of indolic compounds after 12 h and reached a maximum of 96.5 μg.mL^-1^ after 60 h of growth. On the other hand, the indolic compounds production by Gdiaa01 and Gdiaa34 mutants reached, respectively, about 0.64 and 0.74 μg.mL^-1^ after 12 h, with maximum levels achieved after 48 h with values up to 3.8 and 3.4 μg.mL^-1^, respectively, which corresponds to a reduction of approximately 96% as compared to wild-type. When grown in LGIP medium without L-tryptophan, the production of indolic compounds remained below of 1.5 μg.mL^-1^ in both PAL5^T^ and the two selected mutants (data not shown).

To identify the indolic compounds produced by PAL5^T^ and the mutants, the supernatants of bacteria grown in LGIP medium with or without L-tryptophan were analyzed by HPLC. Production of IAA, IPyA, ILA, and anthranilate was observed for the PAL5^T^ strain only when it was grown in medium supplemented with tryptophan (**Supplementary Figure 1**). The compounds IAM, IAN, tryptamine, indole-3-propionate, indole-3-ethanol, and indole were not detected in the supernatant of the wild-type strain neither in the mutants. Because of its instability, two tautomers of IPyA may be observed during chromatography experiments (Schwarz and Bitancourt, 1960). In our experiments, two forms of IPyA were detected with retention times of 18.56 and 27.39 min. ILA was detected in the culture supernatant of PAL5^T^ strain in up to 35.8 μg.mL^-1^ after 60 h of growth, when total indolic compounds produced by PAL5^T^ strain summed around of 117.7 μg.mL^-1^ (**Table 2**). Both mutants produced low levels of IPyA and ILA, however, no IAA could be detected in the supernatant cultures of mutant strains; in addition, the initial supplemented L-tryptophan remained in the culture medium even after 60 h of growth (**Table 2**). In contrast to PAL5^T^, the concentration of total indolic compounds in the supernatant of mutant strains Gdiaa01 and Gdiaa34 were as low as of 2.9 and 4.7 μg.mL^-1^, respectively, after 60 h of growth in the presence of tryptophan. The sum of absolute values of indolic compounds in the supernatants of bacterial cultures differed when determined by HPLC and Salkowski assays; however, data expressed as % of the indolic compounds produced by the wild strain showed similar values. Despite of the small differences between the two mutants, which may be due to minor disparities in the growth at this sampling point, HPLC and Salkowski data confirmed that the levels of indolic compounds were similarly reduced in both mutants (**Figure 1**).

**Table 2:** Quantitative High Performance Liquid Chromatography (HPLC) analysis of indolic compounds produced by PAL5^T^ and mutants of *G. diazotrophicus* grown in LGIP medium with L-tryptophan.

Indolic compounds	PAL5^T^		Gdiaa01		Gdiaa34	
			
	16 h	60 h	16 h	60 h	16 h	60 h
**Production of indolic compounds (μg.mL^-1^)^a^**						
Tryptophan	24.9	6.4	29.4	31.2	30.3	25.5
Anthranilate	4.0	3.4	nd	nd	nd	nd
Indole-3-acetic acid	5.7	13.1	nd	nd	nd	nd
Indole-3-lactate	nd	35.8	nd	0.7	nd	2.7
Indole-3-pyruvate^b^	38.5	nd	1.9	1.9	1.7	1.0
Indole-3-pyruvate^b^	36.6	65.4	nd	0.3	nd	1.0
Total HPLC^c^	84.8	117.7	1.9	2.9	1.7	4.7
Salkowski^d^	27.35	96.54	0.78	3.27	0.87	3.43
% of wild-type (HPLC)	100	100	2.2	2.4	2.0	3.9
% of wild-type (Salkowski)	100	100	2.9	3.4	3.2	3.6


*^a^Identification and quantification was based on retention time and area of each peak according calibration curve of indolic compound standards (“Material and Methods” section); ^b^Represent the two tautomers of indole-3-pyruvate; ^c^Sum of indolic compounds detected (single values in μg.mL^-1^); ^d^Salkowski quantification as presented in the **Figure 1**; The % was calculated by total of indolic compounds compared to PAL5^*T*^ strain. nd – not detected.*

**FIGURE 2:**
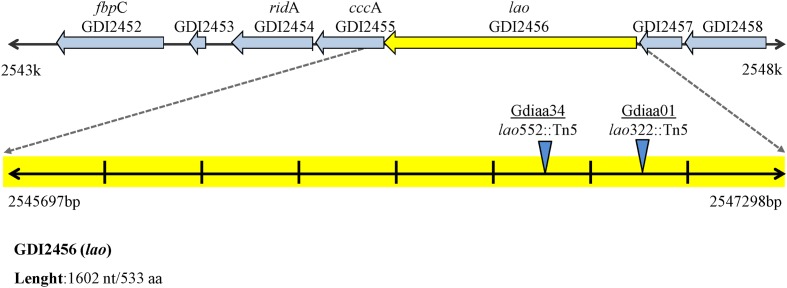
**Genomic context of L-amino acid oxidase gene (*lao*) of *G. diazotrophicus*.** Blue triangles indicate the transposon insertion sites of Gdiaa01 and Gdiaa34 mutants. The *rid*A, *ccc*A, and *lao* genes that form the operon are indicated. Figure adapted from Rhizobase (http://genome.microbedb.jp/rhizobase/GDIA/genes/GDI2456).

**FIGURE 3:**
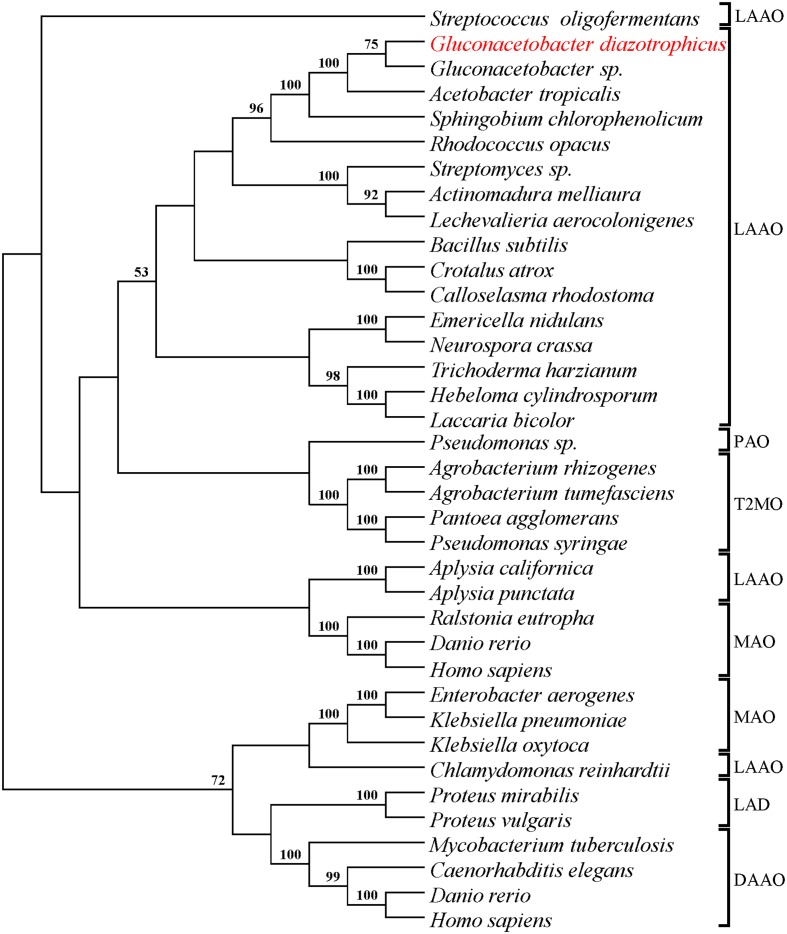
**Condensed Neighbor-Joining tree (topology only) of the L-amino acid oxidase from *G. diazotrophicus* and others validate amine oxidases.** A total of 213 aligned sites were used to compute JTT distance matrix with gamma correction (parameter *a* = 2.55). Bootstrap values higher than 50% are presented on the branches (1000 iterations). Amino acid sequences (**Supplementary Table 1**) of the following proteins were used in phylogenetic analysis: L-amino-acid oxidase (LAAO), L-phenylalanine oxidase (PAO), L-amino acid deaminase (LAD), D-amino-acid oxidase (DAAO), monoamine oxidase (MAO), and tryptophan 2-monooxygenase (T2MO).

The number and position of transposon insertion sites were determined by Southern analysis and sequencing of the transposon-flanking DNA. These analyses revealed that Gdiaa01 and Gdiaa34 mutants had single Tn5 insertions, which are located at different sites within the same open reading frame (GDI2456). In Gdiaa01, Tn5 was inserted at 322 bp of GDI2456, while in Gdiaa34, it was inserted at 522 bp (**Figure 2**), and thus indicating that disruption of this open reading frame resulted in lower capability of IAA biosynthesis by *G. diazotrophicus*. The sequence of 1,602 bp of GDI2456 is predicted to encode an L-amino acid oxidase (LAAO; EC 1.4.3.2) of 533 amino acids, with a calculated molecular mass of 58 kDa. Local alignment of the LAAO predicted amino acid sequence (GenBank accession CAP56399) with the validated LAAO of *Rhodococcus opacus* (GenBank accession AAL14831.1; Geueke and Hummel, 2002) showed a high coverage (97%) between the two sequences. Although the amino acid sequence level identity was rather low (34%), tertiary structure prediction of *G. diazotrophicus* LAAO (**Supplementary Figure 2**) showed a very high structural conservation when compared to the *R. opacus* LAAO model (RMSD score 0.53). Further sequence analyses revealed that both LAAO’s contain a large amine oxidase domain (pfam01593), a FAD/NAD(P)-binding domain and an N-terminal twin-arginine translocation signal of 38 amino acids. The proteins are predicted to have at least three transmembrane domains. In addition, the phylogenetic analysis with other amine oxidases revealed that LAAO of *G. diazotrophicus* shares a clade with other known L-amino acid oxidases from bacteria, actinobacteria, fungi, and the well-characterized LAAO of snakes (**Figure 3**).

**FIGURE 4:**
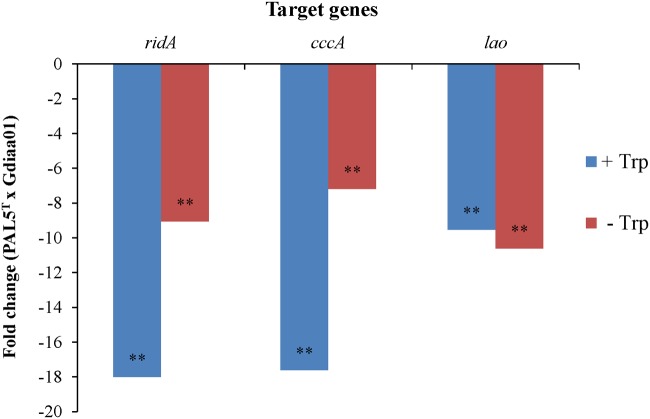
**Relative expression levels of the genes *rid*A, *ccc*A, and *lao* of *G. diazotrophicus* PAL5^T^ strain and mutant Gdiaa01.** Bacteria were grown in modified LGIP broth with or without 100 μg.mL^-1^ of tryptophan. Relative expression and statistical significance were determined from three independent biological samples, each one measured in triplicate by REST-MCS^©^ software, with primer efficiency correction and 2000 randomized interactions. Data are presented as fold change in expression levels of each gene between PAL5^T^ and Gdiaa01 grown in the presence or absence of tryptophan. Gene expression estimates were normalized to the expression of the *rpoD* reference gene. ^∗∗^denotes a significant difference at *p* < 0.001 in expression levels of each gene in PAL5^T^ versus Gdiaa01.

Genomic context analyses (**Figure 2**) revealed that *lao* is adjacent to ORFs GDI2455 (*ccc*A) and GDI2454 (*rid*A), which are predicted to encode, respectively, a putative cytochrome C and an RidA (reactive intermediate deaminase A) protein. According to the OperonDB database, the genomic organization of *lao* cluster is conserved in other bacteria such as *Caulobacter crescentus* and *Xanthomonas* species. The genes *lao* and *ccc*A indeed co-occur in the same direction in other five genomes while *lao* and *rid*A co-occur together in another 19 genomes, with an estimated confidence value of 60% that these genes are expressed as components of the same operon. In accordance with these results, RT-qPCR showed that relative expression of the three genes was significantly (*p* < 0.001) lower in the Gdiaa01 mutant compared to PAL5^T^ strain (**Figure 4**). In the presence of tryptophan, the relative expression of *ridA. cccA*, and *lao* transcripts was, respectively, 18.0, 17.6, and 9.5-fold lower in the Gdiaa01 mutant than in PAL5^T^ strain. In the absence of tryptophan, the transcript levels of *ridA. cccA*, and *lao* was approximately 7- to 10-fold lower in Gdiaa01 than in PAL5^T^ strain. This data further supports that expression of *ridA. cccA*, and *lao* is required for IAA biosynthesis via the IPyA pathway.

**FIGURE 5:**
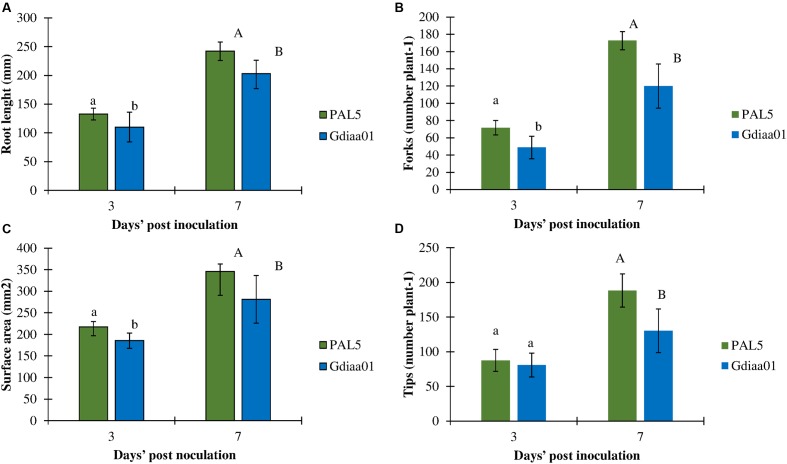
**Root development of rice inoculated with PAL5^T^ and mutant Gdiaa01.** **(A)** Root length, **(B)** surface area, **(C)** number of branches, and **(D)** number of tips of rice roots after 3 and 7 days’ post inoculation (DPI). Data represent the mean ± standard error (SE) of four biological replicates. Different lower case and capital letters above the error bars indicate a significant difference between strains at three and seven DPI, respectively, according pairwise *t*-test at *p* ≤ 0.05.

The effect of the mutation in the *G. diazotrophicus lao* gene cluster and the consequent reduction of IAA production was also investigated regarding the plant growth-promotion effect, defined here as the increase in the early developmental stage of rice roots due to inoculation. The results showed that plants inoculated with PAL5^T^ had significantly greater root development than those plants inoculated with the mutant Gdiaa01 (**Figure 5**). The modification in root architecture was observed 3 days after inoculation, where plants inoculated with the mutant showed decreases in root length (-18%), root surface area (-16%), number of branches (-23%), and number of root tips (-21%) as compared to plants inoculated with the wild-type. These effects were even more pronounced after 7 days of inoculation: the length of roots, the number of branches and tips were, respectively, 22, 29, and 32% lower in plants inoculated with the mutant than in plants inoculated with wild-type.

## Discussion

The biosynthesis of IAA by the IPyA pathway is mediated by the key protein indole-3-pyruvate decarboxylase (IPDC). The IPDC is encoded by *ipd*C gene and catalyzes the decarboxylation of IPyA to indole-3-acetaldehyde (IAAld) intermediate, which is then further oxidized to IAA (Spaepen et al., 2007; Spaepen and Vanderleyden, 2011; Patten et al., 2013). Based on detection of ILA and indole-3-ethanol (tryptophol), products of the reversible reduction of IPyA and IAAld, respectively, it was previously suggested that this pathway is responsible for production of IAA in *G. diazotrophicus* (Fuentes-Ramirez et al., 1993; Lee et al., 2004). However, searches for the *ipd*C gene in the genome of *G. diazotrophicus* PAL5^T^ resulted in only one positive hit, a *pdc* gene that encodes a pyruvate decarboxylase (PDC). Recent studies with this PDC showed that IPyA was not used as a substrate (van Zyl et al., 2014), which excludes this decarboxylase as the enzyme responsible for IAA production in *G. diazotrophicus*.

In our work, we elucidated important characteristics about the IAA biosynthesis in this endophytic diazotrophic bacterium. Our first finding was to confirm that *G. diazotrophicus* synthesize IAA by the IPyA pathway when tryptophan is available in the culture medium. The compounds identified in the supernatant of PAL5^T^ (IPyA and ILA) belong to the IPyA pathway, reinforcing that this is the principal route for IAA biosynthesis in *G. diazotrophicus*. An observation consistent with this conclusion is that the intermediates of other pathways (tryptamine, IAM, or IAN) were not detected in our analysis neither in previous studies (Fuentes-Ramirez et al., 1993; Lee et al., 2004). Although IAAld is an intermediate of IPyA pathway (Spaepen et al., 2007; Spaepen and Vanderleyden, 2011; Patten et al., 2013), it was not detected by previous HPLC analyses, although the indole-3-ethanol, the product of IAAld reduction, has been detected by thin-layer chromatography (Lee et al., 2004). IAAld as well as IPyA are very unstable compounds and can to be degraded spontaneously, making difficult their detection (Koga et al., 1991). In plants, IAA can be directly produced from IPyA by a YUC flavin-containing monooxygenases (Zhao, 2012). So, it is possible that the same occurs in *G. diazotrophicus* and therefore, further analyses are needed to clarify whether IAAld is an intermediate in IAA biosynthesis by *G. diazotrophicus*. Interestingly, anthranilate was detected only in PAL5^T^ supernatant although in lower levels if compared to the concentration of other indolic compounds identified. Anthranilate can be produced from tryptophan degradation, e.g., via kyunerine pathway (Kurnasov et al., 2003) or be derived from the IAA catabolism (Leveau and Gerards, 2008). Considering lack of gene homologous to the kyunerine pathway in the *G. diazotrophicus* PAL5^T^ genome, as well as the presence of anthranilate in the supernatant of wild-type strain but not in the mutant strains, the existence of an IAA catabolism pathway in *G. diazotrophicus* cannot be discarded as the source of anthranilate, although this statement requires further investigation.

A second novel finding obtained in this work was the identification of a gene cluster involved in IAA biosynthesis by *G. diazotrophicus.* As shown, interruptions of the *lao* gene occurred in two independent mutants (Gdiaa01 and Gdiaa34) resulting in a reduction of 100% of IAA and up to 95% in the production of total indolic compounds. Sequence analysis using different bioinformatics tools revealed that the interrupted gene is predicted to encode an L-amino acid oxidase. In *G. diazotrophicus* PAL5, the *lao* gene constitutes a cluster together with *ccc*A and *rid*A genes, which also showed reduced expression levels in the mutant strains when compared to the type strain, regardless the presence of tryptophan in the culture media. This finding suggests that these genes constitute an operon and that Tn5 insertion affected not only the transcription of the *lao* gene but also of the *ccc*A and *rid*A genes. Interestingly, the *lao* expression in the mutant strain was not inhibited to the same extent as that observed for the *cccA* and *ridA* genes (**Figure 4**). Variations in the regulation of genes from a same operon is known to be related to the presence of internal promoter sequences on the operon, that allows independent regulation for each gene by different transcription factors (Ishihama, 2010; Napolitano et al., 2013). Recently, Yu et al. (2015) reported that the LAAO of *Pseudoalteromonas* sp. is regulated at both the transcription and post-transcriptional levels, since mutations in regulator genes abolished the activity of LAAO. The bioinformatic analyses of *lao* operon in *G. diazotrophicus* revealed at least six promoter sequences suitable to bind different transcription factors (data not shown). Nevertheless, the regulation of the genes of *lao* cluster in *G. diazotrophicus* is beyond the scope of this work and deserves further studies.

Although complementation and expression studies may help to define the molecular function of each gene within the *lao* cluster, our findings suggest that all three genes may be involved in IAA biosynthesis. Therefore, we hypothesize that cytochrome C and the RidA proteins possibly work together with the LAAO in biosynthesis of IAA. Previous studies with these proteins support this hypothesis. LAAO (EC 1.4.3.2) is a flavoenzyme that catalyzes the stereo-specific oxidative deamination of L-amino acids to their corresponding α-ketoacids with a concurrent release of NH_4_^+^ and H_2_O_2_. When H_2_O_2_ is not degraded by catalase, it can cause the decarboxylation of the α-ketoacid to the corresponding carboxylic acid (Pawelek et al., 2000; Yu and Qiao, 2012). LAAO has been described in many different organisms as snakes, algae, fungi, mollusks, and also in diverse bacterial species (Yu and Qiao, 2012; Pollegioni et al., 2013; Hossain et al., 2014). The well-characterized LAAO of *R. opacus* exhibits a very broad substrate specificity oxidizing 39 out of 43 tested L-amino acids, including L-tryptophan (Geueke and Hummel, 2002). In *Lechevalieria aerocolonigenes*, for instance, the L-amino acid oxidase RebO is a key enzyme in the synthesis of rebeccamycin, a tryptophan-derived indolocarbazole (Nishizawa et al., 2005). RebO is known as a tryptophan oxidase and recently, the overexpression of RebO in *Arabidopsis* affected the auxin biosynthesis and plant development and also, it rescues all aspects of developmental defects displayed in mutants of *Arabidopsis* that are defective in producing IPyA (Gao et al., 2016). In *Chromobacterium violaceum*, the flavoenzyme VioA, an L-amino acid oxidase highly specific for L-TRP, is involved in the synthesis of the purple chromobacterial pigment violacein (Balibar and Walsh, 2006). In the basidiomycete *Coprinus*, a membrane-bound L-tryptophan oxidase (TOD) catalyzes the simultaneous oxidative deamination and oxidative decarboxylation of L-TRP to produce IPyA and IAM, respectively (Furuya et al., 2000). Therefore, it is possible that LAAO of *G. diazotrophicus* can catalyze the conversion of L-tryptophan to IPyA, the first step of IAA biosynthesis by IPyA pathway, a role that needs to be further confirmed. Despite of LAAO catalyzes the production of IPyA from L-tryptophan, only recently this activity was related to IAA biosynthesis (Gao et al., 2016). This step (TRP to IPyA) is generally attributed to activity of unspecific amino acid aminotransferases (AATs). As observed in HPLC (**Supplementary Figure 1**), minor amounts of IPyA were still detected in mutant supernatants. Since aromatic AAT activity has been reported in *G. diazotrophicus* earlier (Pedraza et al., 2004), it may be the responsible for the low levels of IPyA production from TRP displayed by both mutants.

In relation to the cytochrome C and RidA proteins, previous studies provide evidences that these proteins may function together with LAAO. It was found earlier in *G. diazotrophicus* that mutations in *ccm* genes, which encode proteins required for cytochrome C maturation, are also involved in IAA synthesis, since the *ccm* mutants produced only 5% of wild-type levels of IAA (Lee et al., 2004; Reis et al., 2007). As suggested by these authors cytochrome C is likely to be an essential component of an IAA biosynthetic enzyme with redox functions in *G. diazotrophicus*. Cytochrome C is an electron transfer protein widely distributed among bacteria that participates in diverse processes such as respiration and H_2_O_2_ scavenging (Thöny-Meyer, 1997; Stevens et al., 2004; Bertini et al., 2006). Also, cytochrome C often interacts with other redox enzymes in which it constitutes an entry/exit point for electrons in the catalytic cycle of the enzyme (Bertini et al., 2006). Since cytochrome C is an electron transfer protein known to interact with other redox proteins, it is possible that cytochrome C plays a role in electron transfer needed for LAAO function as earlier proposed (Lee et al., 2004), but this needs further investigation. About the RidA protein, a recent study found that it hydrolyzes reactive imine/enamine intermediates produced by L-amino acid oxidases to its respective α-ketoacids in this way preventing any cellular damage caused by the increase on its concentration (Niehaus et al., 2015). An enzyme complex constituted of LAAO (catalytic Mα subunit) and a RidA protein (non-catalytic Mβ subunit) was earlier observed in the periplasmic space of the green algae *Chlamydomonas reinhardtii*, in which it probably acts scavenging NH_4_^+^ from extracellular L-amino acids (Vallon et al., 1993; Merchant et al., 2007). Similarly, ours analyzes also suggested that predicted LAAO is probably located in periplasmic space of *G. diazotrophicus*, since it carries three transmembrane domains and a signal peptide typical of proteins exported by Tat secretion system, which is known to export proteins to the periplasm or periplasmic face of the bacterial membrane (Berks et al., 2003). Membrane-bound LAAOs have been reported to interact with a respiratory electron transport chain, resulting in release of water instead of H_2_O_2_ (Hossain et al., 2014). So, further studies are needed to confirm the cellular localization of predicted LAAO even as, the possible collaboration between cytochrome C, RidA, and LAAO proteins in biosynthesis of IAA by *G. diazotrophicus.*

Plant growth promotion by *G. diazotrophicus* is attributed to biological nitrogen fixation, mineral nutrient solubilization, and production of phytohormones, mainly auxins (Pedraza, 2008; Eskin et al., 2014; Reis and Teixeira, 2015). In our study, an interruption of *lao* gene and the consequent reduction in IAA biosynthesis ability lead to the suppression of growth-promoting effect on development of rice roots. Inoculation of rice with Gdiaa01 mutant resulted in shorter roots, smaller root area, and lower numbers of branches and tips when compared to plants inoculated with the wild-type. Inoculation studies with mutants of *G. diazotrophicus* have shown that both nitrogen fixation and IAA biosynthesis are essential for improving sugarcane growth, and the inoculation of micropropagated sugarcane plants with *G. diazotrophicus* PAL5 mutants deficient in IAA production (*ccm*C), nitrogen fixation (*nif*D), or in both abilities (*ccm*C-*nif*D double mutant) significantly reduced the shoot and root dry mass and did not differ from non-inoculated plants grown both under N-deficiency or N-supply conditions (Lee, 2001; Reis et al., 2007). Our results are in agreement with those studies, confirming the importance of the bacterial phytohormone IAA in promoting the plant growth by *G. diazotrophicus*. Considering that IAA can act as a reciprocal signaling molecule in microbe-plant interactions (Spaepen and Vanderleyden, 2011), studies on the expression of the *G. diazotrophicus lao* gene cluster during the endophytic colonization of plants should be further investigated.

## Conclusion

Our study demonstrates that *G. diazotrophicus* produces IAA via the IPyA pathway and provides evidence for the involvement of an L-amino acid oxidase gene cluster, constituted by *lao. ccc*A, and *rid*A genes, in the biosynthesis of this phytohormone. To the best of our knowledge, this is the first work that indicates the involvement of an L-amino acid oxidase gene cluster in bacterial IAA biosynthesis. According to the roles performed by LAAO and by cytochrome C and RidA proteins as described in literature, we hypothesize that the predicted LAAO works in first step of L-tryptophan metabolism by the IPyA pathway, it can be assisted in its redox reactions by predicted cytochrome C and RidA proteins. Further studies are needed to establish the molecular function of each gene in IAA biosynthesis and their interaction with plants.

## Author Contributions

JB: Corresponding author; results analysis, reviewed the manuscript. ER: Experimental design; results analysis, wrote the manuscript. CdP: Experimental design; performed experiments; results analysis. PG: Experimental design; performed experiments; results analysis. EI: Bioinformatics analyses; wrote the manuscript. JdA: Results analysis, reviewed the manuscript. LR: Results analysis, reviewed the manuscript. AdO: Bioinformatics analyses; wrote the manuscript. MV: Experimental design; results analysis, wrote the manuscript.

## Conflict of Interest Statement

The authors declare that the research was conducted in the absence of any commercial or financial relationships that could be construed as a potential conflict of interest.
