# Supplementary material for: Identification of Genes Involved in Indole-3-Acetic Acid Biosynthesis by Gluconacetobacter diazotrophicus PAL5 Strain Using Transposon Mutagenesis
Source: Front Microbiol. 2016 Oct 7;7:1572. doi: 10.3389/fmicb.2016.01572 (PMC5053998; doi:10.3389/fmicb.2016.01572)
Supplement: Supplementary file 1 [file Table_1.DOCX]

Supplementary Material

**Identification of genes involved in indole-3-acetic acid biosynthesis by *Gluconacetobacter diazotrophicus* PAL5 strain using transposon mutagenesis**

**Elisete Pains Rodrigues, Cleiton de Paula Soares, Patrícia Gonçalves Galvão, Eddie Luidy Imada, Jean Luiz Simões de Araújo, Luc Felicianus Marie Rouws, André Luiz Martinez de Oliveira, Márcia Soares Vidal, José Ivo Baldani3***

*** Correspondence:** José Ivo Baldani: [ivo.baldani@embrapa.br](mailto:ivo.baldani@embrapa.br)

**Suplementary tables:**

**Suplementary table 1. Sequences used for phylogenetic analysis of the LAAO**

| **Taxonomic group** | **Species** | **Genbank accession number** |
| --- | --- | --- |
| Firmicutes | *Streptococcus oligofermentans* | [gb\|ACA52024.1\|](http://www.ncbi.nlm.nih.gov/protein/169260271?report=genbank&log$=protalign&blast_rank=1&RID=RH62W051015) |
|  | *Bacillus subtilis* | [sp\|O34363.2\|YOBN_BACSU](http://www.ncbi.nlm.nih.gov/protein/221223004?report=genbank&log$=protalign&blast_rank=2&RID=RH5W45ZC014) |
| Proteobacteria | *Gluconacetobacter diazotrophicus* | [CAP56399](http://bacteria.ensembl.org/gluconacetobacter_diazotrophicus_pa1_5/Transcript/ProteinSummary?db=core;g=GDI2456;r=Chromosome:2545697-2547298;t=CAP56399) |
|  | *Gluconacetobacter* sp. | [gb\|EGG78812.1\|](http://www.ncbi.nlm.nih.gov/protein/329762325?report=genbank&log$=protalign&blast_rank=1&RID=RH4J7NDS014) |
|  | *Acetobacter tropicalis* | [dbj\|GAA08193.1\|](http://www.ncbi.nlm.nih.gov/protein/338752162?report=genbank&log$=protalign&blast_rank=1&RID=RH4PBC62014) |
|  | *Sphingobium chlorophenolicum* | [gb\|AEG51547.1\|](http://www.ncbi.nlm.nih.gov/protein/334104124?report=genbank&log$=protalign&blast_rank=1&RID=RH4T7KYX014) |
|  | *Pseudomonas* sp. | [dbj\|BAD66877.1\|](http://www.ncbi.nlm.nih.gov/protein/54649976?report=genbank&log$=protalign&blast_rank=1&RID=RH4S9WJA015) |
|  | *Agrobacterium rhizogenes* | [gb\|AAA22080.1\|](http://www.ncbi.nlm.nih.gov/protein/385072?report=genbank&log$=protalign&blast_rank=1&RID=RH4XCPB5014) |
|  | *Agrobacterium tumefaciens* | [sp\|P0A3V2.1\|TR2M_AGRT4](http://www.ncbi.nlm.nih.gov/protein/61248670?report=genbank&log$=protalign&blast_rank=1&RID=RH4YXFFZ015) |
|  | *Pantoea agglomerans* | [gb\|AAC17187.1\|](http://www.ncbi.nlm.nih.gov/protein/499582?report=genbank&log$=protalign&blast_rank=1&RID=RH54B3Y5015) |
|  | *Pseudomonas syringae* | [sp\|P06617.1\|TR2M_PSESS](http://www.ncbi.nlm.nih.gov/protein/136116?report=genbank&log$=protalign&blast_rank=1&RID=RH55VBM1014) |
|  | *Ralstonia eutropha* | [ref\|YP_725346.1\|](http://www.ncbi.nlm.nih.gov/protein/113866857?report=genbank&log$=protalign&blast_rank=1&RID=RH58FXVR015) |
|  | *Enterobacter aerogenes* | [dbj\|BAA01060.1\|](http://www.ncbi.nlm.nih.gov/protein/216723?report=genbank&log$=protalign&blast_rank=1&RID=RH5BPE2901R) |
|  | *Klebsiella pneumoniea* | [sp\|P49250.1\|AMO_KLEAE](http://www.ncbi.nlm.nih.gov/protein/1351922?report=genbank&log$=protalign&blast_rank=1&RID=RH5DDX2J014) |
|  | *Klebsiella oxytoca* | [sp\|P80695.2\|AMO_KLEOK](http://www.ncbi.nlm.nih.gov/protein/387912822?report=genbank&log$=protalign&blast_rank=1&RID=RH5CTMT5015) |
|  | *Proteus mirabilis* | [gb\|ACD36582.1\|](http://www.ncbi.nlm.nih.gov/protein/187765513?report=genbank&log$=protalign&blast_rank=1&RID=RH5MX6EC014) |
|  | *Proteus vulgaris* | [dbj\|BAA90864.1\|](http://www.ncbi.nlm.nih.gov/protein/7007412?report=genbank&log$=protalign&blast_rank=1&RID=RH5MRRSZ01R) |
| Actinobacteria | *Rhodococcus opacus* | [gb\|AAL14831.1\|](http://www.ncbi.nlm.nih.gov/protein/18026226?report=genbank&log$=protalign&blast_rank=1&RID=RH4BC67V01R) |
|  | *Streptomyces* sp. | [dbj\|BAC55210.1\|](http://www.ncbi.nlm.nih.gov/protein/27753576?report=genbank&log$=protalign&blast_rank=1&RID=RH4BY2S1014) |
|  | *Actinomadura melliaura* | [gb\|ABC02789.1\|](http://www.ncbi.nlm.nih.gov/protein/83320224?report=genbank&log$=protalign&blast_rank=1&RID=RH48N7CC015) |
|  | *Lechevalieria aerocolonigenes* | [dbj\|BAC10674.1\|](http://www.ncbi.nlm.nih.gov/protein/22535501?report=genbank&log$=protalign&blast_rank=1&RID=RH44RGXU01R) |
|  | *Mycobacterium tuberculosis* | [sp\|O07727.1\|DAO_MYCTU](http://www.ncbi.nlm.nih.gov/protein/81668903?report=genbank&log$=protalign&blast_rank=1&RID=RH3XD66V015) |
| Viperidae | *Crotalus atrox* | [sp\|P56742.2\|OXLA_CROAT](http://www.ncbi.nlm.nih.gov/protein/124106294?report=genbank&log$=protalign&blast_rank=1&RID=RH3W08GC014) |
|  | *Calloselasma rhodostoma* | [sp\|P81382.2\|OXLA_AGKRH](http://www.ncbi.nlm.nih.gov/protein/20141785?report=genbank&log$=protalign&blast_rank=1&RID=RH3W95J1014) |
| Ascomycota | *Emericella nidulans* | [gb\|AAT84085.2\|](http://www.ncbi.nlm.nih.gov/protein/56757832?report=genbank&log$=protalign&blast_rank=1&RID=RH3M12HT01R) |
|  | *Neurospora crassa* | [gb\|EAA32442.1\|](http://www.ncbi.nlm.nih.gov/protein/28923226?report=genbank&log$=protalign&blast_rank=1&RID=RH3N7JJW015) |
|  | *Trichoderma harzianum* | [gb\|ADD91592.2\|](http://www.ncbi.nlm.nih.gov/protein/313664888?report=genbank&log$=protalign&blast_rank=1&RID=RH3TD1VV01R) |
| Basiodiomycota | *Hebeloma cylindrosporum* | [gb\|ADM80414.1\|](http://www.ncbi.nlm.nih.gov/protein/306022302?report=genbank&log$=protalign&blast_rank=1&RID=RH2X75WH01R) |
|  | *Laccaria bicolor* | [tpg\|DAA34975.1\|](http://www.ncbi.nlm.nih.gov/protein/356640139?report=genbank&log$=protalign&blast_rank=1&RID=RH2YCNKM015) |
| Mollusca | *Aplysia californica* | [gb\|AAT12273.1\|](http://www.ncbi.nlm.nih.gov/protein/47156785?report=genbank&log$=protalign&blast_rank=1&RID=RH2NH2WB014) |
|  | *Aplysia punctata* | [gb\|AAR14185.1\|](http://www.ncbi.nlm.nih.gov/protein/38230180?report=genbank&log$=protalign&blast_rank=2&RID=RH2NH2WB014) |
| Actinopterygii | *Danio rerio* | [gb\|AAH70013.1\|](http://www.ncbi.nlm.nih.gov/protein/47124956?report=genbank&log$=protalign&blast_rank=1&RID=RH3EV288014) |
|  | *Danio rerio* | [gb\|AAH66686.1\|](http://www.ncbi.nlm.nih.gov/protein/45219748?report=genbank&log$=protalign&blast_rank=1&RID=RH3E7X0X01R) |
| Mammalia | *Homo sapiens* | [sp\|P21397.1\|AOFA_HUMAN](http://www.ncbi.nlm.nih.gov/protein/113978?report=genbank&log$=protalign&blast_rank=1&RID=RH21TBWC015) |
|  | *Homo sapiens* | [sp\|P14920.3\|OXDA_HUMAN](http://www.ncbi.nlm.nih.gov/protein/25453448?report=genbank&log$=protalign&blast_rank=1&RID=RH24FZXW01R) |
| Chlorophyta | *Chlamydomonas reinhardtii* | [gb\|EDP03066.1\|](http://www.ncbi.nlm.nih.gov/protein/158277297?report=genbank&log$=protalign&blast_rank=1&RID=RH356J9F015) |
| Nematoda | *Caenorhabditis elegans* | [dbj\|BAF34313.1\|](http://www.ncbi.nlm.nih.gov/protein/115510996?report=genbank&log$=protalign&blast_rank=1&RID=RH3B8JN5015) |
