# Supplementary material for: Identification of Genes Involved in Indole-3-Acetic Acid Biosynthesis by Gluconacetobacter diazotrophicus PAL5 Strain Using Transposon Mutagenesis
Source: Front Microbiol. 2016 Oct 7;7:1572. doi: 10.3389/fmicb.2016.01572 (PMC5053998; doi:10.3389/fmicb.2016.01572)
Supplement: Supplementary file 2 [file Presentation_1.PDF]

Supplementary Material

Identification of genes involved in indole-3-acetic acid biosynthesis by *Gluconacetobacter diazotrophicus* PAL5 strain using transposon mutagenesis

Elisete Pains Rodrigues, Cleiton de Paula Soares, Patrícia Gonçalves Galvão, Eddie Luidy Imada, Jean Luiz Simões de Araújo, Luc Felicianus Marie Rouws, André Luiz Martinez de Oliveira, Márcia Soares Vidal, José Ivo Baldani3*

*** Correspondence:** José Ivo Baldani: ivo.baldani@embrapa.br

Suplementary Figures

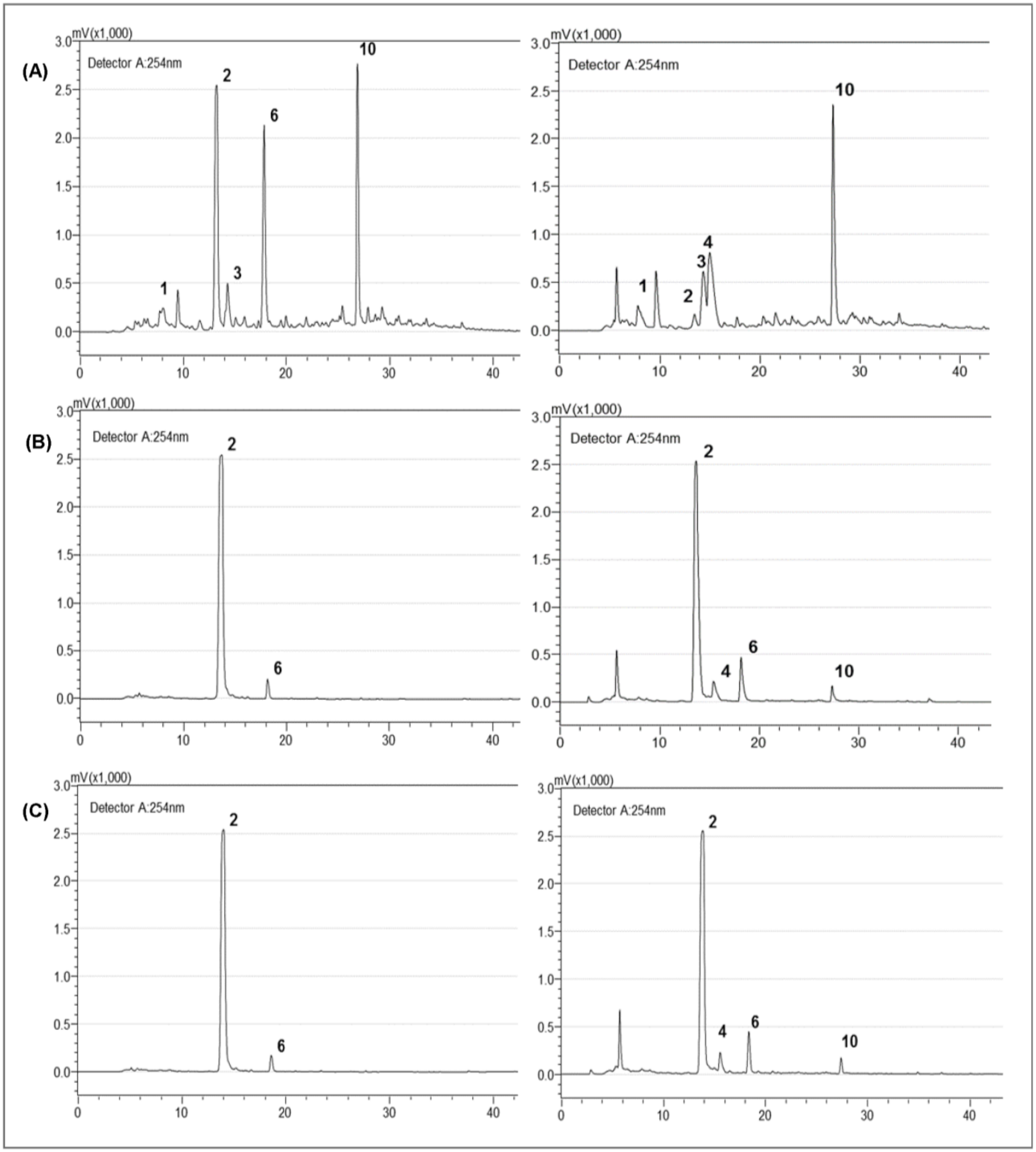

Supplementary Figure 1. HPLC of indolic compounds of PAL5^T (A) and mutants Gdiaa01 (B) and Gdiaa34 (C). Bacteria were grown in LGIP medium with 100 µg.mL^{-1} of L-tryptophan. Supernatant was collected after 16 and 60 hours of growth and indolic compounds were analyzed by HPLC on a C18 column with a gradient of methanol and phosphate buffer. Data shown in left panels are from 16h cultures and right panels are from 60h cultures. Peaks represent anthranilate (1), L-tryptophan (2), indole-3-acetate (3), indole-3-lactate (4) and indole-3-pyruvate tautomers (6 and 10).

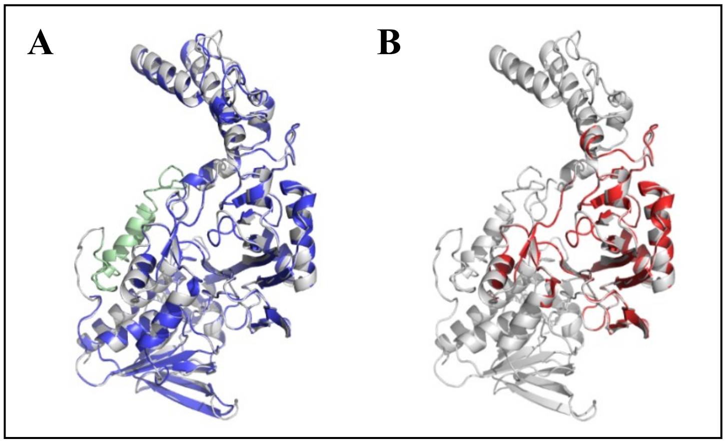


```
AAL14831        -------MAFTRRSFMKGLGATGGAGLAYGAMSTLGLAPSTAAPARTFQPLAAG 47
CAP56399        MTNAFRRPAATRRQMLTRIGILGGSAALYQAMTSLGHAAGTDFSG--------P 46
                        * ***.::. :*  **:.  * **::** *..*  ..
AAL14831        DLIGKVKGSHSVVVLGGGPAGLCSAFELQKAGYKVTVLEARTRPGGRVWTARGG 101
CAP56399        PVLTGAKRGTRVLVLGAGLAGMLAAYELRKAGYQVRILEFQNRSGGRNISLRGG 100
                 ::  .* .  *:***.* **: :*:**:****:* :** :.*.***  : ***
AAL14831        SEETDLSGETQKCTFSEGHFYNVGATRIPQSHI-TLDYCRELGVEIQGFGNQNA 154
CAP56399        DTLTELGGATQKVQFAPGNYINPGPWRIPYHHQGLLHYCKAFGVELEPFVELNH 154
                .  *:*.* ***  *: *:: * *. ***  *   *.**: :***:: * : *
AAL14831        NTFVNYQSDTSLSGQSVTYRAAKADTFGYMSELLKKATDQGALDQVLSREDKDA 208
CAP56399        NSWLHTS--DAFGGKAVRYREFASDFNGFTAELLSKAIEQHKLDDVVSADERAH 206
                *:::: .   ::.*::* **   :*  *: :***.** :*  **:*:* :::
AAL14831        LSEFLSDFGDLSDDGRYLG----SSRRGYDSEPGAGLN-FGTEKKPFAMQEVIR 258
CAP56399        VATAMREWGGLDASGAYRKGVTSSLRRGFAKPQGGGVDGAPIPSDTFARGDVMR 260
                ::  : ::*.*. .* *      * ***: .  *.*::     ...**  :*:*
AAL14831        SGIGRNFSFDFGYDQAMMMFTPVGGMDRIYYAFQDRIGTDNIVFGAEVTSMKNV 311
CAP56399        SGLWQWMAFHERLDMQTTMFQPVGGMDQIGKGFTRQV-HDLITLNCKVSSIHQD 314
                **: : ::*.   *    ** ******:*  .*  ::  * *.:..:*:*:::
AAL14831        SEGVTVEYT---AGGSKKSITADYAICTIPPHLVGRLQNNLPGDVLTALKAAKP 362
CAP56399        DHGVTVTYDDMAHGGAVRQAQADYCVCTIPLSVLSQLDIQVSGPLKAAIAAVPY 367
                ..**** *     **: :.  ***.:****  ::.:*: ::.* : :*: *.
AAL14831        SSSGKLGIEYSRRWWETEDRIYGGASNTDKDISQIMFP-YDHYNSDRGVVVAYY 416
CAP56399        ASSVKLGLEFNRRFWEEDDQIYGGISFTDQPISQISYPSHGYFSNGPAVLLGGY 421
                :** ***:*:.**:** :*:**** * **: **** :* :.::... .*::. *
AAL14831        SSGKRQEAFESLTHRQRLAKAIAEGSEIHGEKYTRDISSSFSGSWRRTKYSESA 469
CAP56399        MFGPAAYDFAGMSPAERIERGLAQGQVIH-PNYRKEFRSGVAVAWSRMPWTLGC 475
                  *     * .::  :*: :.:*:*. **  :* ::: *..: :* *  :: ..
AAL14831        WANWAGSGGSHGGAATPEYEKLLEPVDKIYFAGDHLSNAIAWQHGALTSARDVV 523
CAP56399        CSMWS------EQARKTHYKTLCEVDNRIVLAGEHASYIGCWQEGAILSSLDAI 522
                 : *:        * ...*:.* *  ::* :**:* *   .**.**: *: *.:
AAL14831        THIHERVAQEA 534
CAP56399        TRLHKRAHGAV 533
                *::*:*.   .
```

Supplementary Figure 2. Tertiary structure prediction of L-amino acid oxidase (LAAO) of *G. diazotrophicus* (Top) and pairwise alignment of protein sequences of *G. diazotrophicus* (CAP56399) and *R. opacus* (AAL14831) (Bottom). (A) Alignment between predicted LAAO tertiary structure of *G diazotrophicus* and *R. opacus* model (blue) showing the signal sequence in green. (B) Alignment between substrate-binding domain (red) of the LAAO of *R. opacus* and predicted LAAO structure of *G. diazotrophicus* (B). At the bottom, the full-length LAAO protein sequences of *G. diazotrophicus* (CAP56399) and *R. opacus* (AAL14831) were aligned with Clustal X version 2.0 (Larkin et al., 2007).
